# N6-methyladenosine demethylase FTO suppressed prostate cancer progression by maintaining CLIC4 mRNA stability

**DOI:** 10.1038/s41420-022-01003-7

**Published:** 2022-04-09

**Authors:** Libin Zou, Wenbin Chen, Xumin Zhou, Taowei Yang, Junqi Luo, Zining Long, Jun Wu, Daojun Lv, Xiangming Mao, Shengren Cen

**Affiliations:** 1 Department of Urology, Zhujiang Hospital, Southern Medical University, Guangzhou, China; 2 Department of Urology, the Third Affiliated Hospital of Guangzhou Medical University, Guangzhou, China

**Keywords:** Prostate cancer, Oncogenesis

## Abstract

The fat mass and obesity-associated protein (FTO) is an N6-Methyladenosine (m6A) demethylase, which has been revealed to play critical roles in tumorigenesis. However, its role in the development and progression of prostate cancer (PCa) remains poorly understood. Here, we aimed to investigate the function and clinical relevance of FTO in PCa. Our results demonstrated that FTO was notably downregulated in PCa tissues compared with the paired normal tissues. In addition, the decreased expression of FTO was correlated with poor prognosis of PCa. Functional experiments showed that depletion of FTO promoted the proliferation and metastasis of PCa both in vitro and in vivo. Conversely, ectopic expression of FTO exhibited the opposite effects. Combined with RNA-sequencing, MeRIP-RT-qPCR, and mRNA stability assays indicated chloride intracellular channel 4(CLIC4) was a functional target of FTO-mediated m6A modification. FTO depletion significantly increased the m6A level of CLIC4 mRNA and then reduced the mRNA stability. In conclusion, our findings suggest that FTO suppresses PCa proliferation and metastasis through reducing the degradation of CLIC4 mRNA in an m6A dependent manner. FTO may be used as a promising novel therapeutic target and prognostic evaluation biomarker for PCa.

## Introduction

Prostate cancer (PCa) is one of the most prevalent tumors and has the second-highest mortality among men, counting for 26% of new incident cases and 11% in estimated death in 2021 [1]. Although surgical resection and androgen deprivation therapy can benefit most patients with local diseases, approximately 20% of primary PCa will develop into a disseminated disease, accounting for the larger portion of PCa-related mortality [2, 3]. Whereas, the underlying molecular mechanism of PCa progress remains elusive, highlighting the urgent needs to investigate new therapeutic targets for PCa.

N6-methyladenosine (m6A) RNA modification, the most predominant post-transcriptional modification of the eukaryotic messenger RNAs [4], has been proved to be widely distributed in the transcriptome and regulated mRNA stability and translation efficiency [5, 6]. m6A modification is reported to be dynamically regulated by m6A methyltransferases (“Writer”) and demethylases (“Eraser”), and the functions of m6A on mRNA fate depends on specific RNA binding proteins (“Reader”), which mediate mRNA translation and stability [5, 7]. Recently, emerging evidence has clarified the essential role of m6A in mediating cancer development [8, 9]. For instance, YTHDF1 can enhance the translation of EIF3C and promote the overall translation products, thereby stimulating proliferation and metastasis of ovarian cancer [10]. IGF2BP1 was shown to promote the development of endometrial cancer by stabilizing the PEG10 mRNA [11]. Moreover, YTHDF2 regulates the degeneration of LHPP and NKX3-1 mRNA and promotes PCa progression through the AKT signaling pathway [12]. However, the regulatory mechanism of m6A in the development of PCa remains largely uncertain.

Here, we focus on m6A modification, especially the role of the demethylase fat mass and obesity-associated protein (FTO) in mediating the development and progression of PCa. As a demethylase, FTO has been reported to play a crucial role in various cancers [13, 14]. For example, FTO can increase the m6A level of HSF1 and then facilitate its decay by YTHDF2, thereby promoting multiple myeloma progression [15]. Ruan et al. reported that FTO plays an anti-metastasis gene role in colorectal cancer in an m6A dependent manner [16]. Thus, FTO have been highlighted as a novel player in tumor development and progression by acting as tumor suppressor or oncogene, depending on the circumstance. However, the specific role of FTO in the development of PCa has not been well characterized.

In the current study, we aim to investigate the expression and clinical relevance of FTO in PCa tissues and cells, and then explore the potential role of FTO in PCa cells in vitro and in vivo. Finally, we further reveal the underlying mechanism of FTO in PCa progression and metastasis. This study might provide a novel insight into the function and mechanism of FTO in PCa pathogenesis.

## Results

### FTO is downregulated in human PCa

**Fig. 1:**
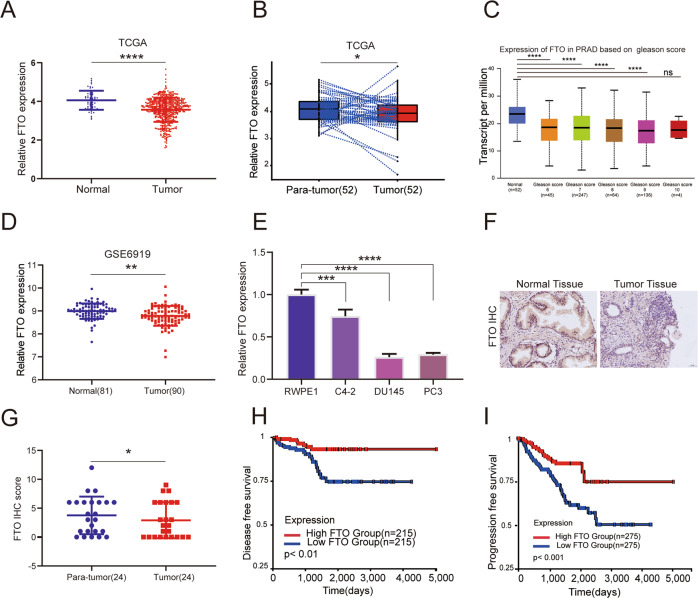
Decreased FTO expression is associated with a poor prognosis of prostate cancer patients. **A**–**C** The expression pattern of FTO in total, paired-samples, and separate Gleason scores were analyzed in PCa tissues and normal controls (log2(TPM + 1), TCGA database). **D** Relative RNA levels of FTO in prostate cancer and normal prostate tissues in GEO datasets. **E** The expression of FTO in various prostate cells was analyzed by qRT-PCR, GAPDH as a loading control. **F**, **G** Representative images of FTO immunohistochemistry analysis in prostate cancer and paraneoplastic tissues; The IHC score of FTO in the paired group; **H**, **I** Kaplan–Meier analysis of PCa patients in TCGA for the correlations between FTO expression and disease-free survival as well as Progression-free interval. *P < 0.05, **P < 0.01, ***P < 0.001, ****P < 0.0001. Error bars, mean ± SD.

To determine the expression pattern of FTO in PCa, we calculated the transcriptomic profiles of PCa tumor samples and adjacent prostate tissues in the TCGA and GEO database (GSE6919), and found that FTO was significantly downregulated in prostate tumors (Fig. 1A–D). Likewise, compared with the normal prostate epithelial cell line(RWPE1), the transcription level of FTO detected in the PCa cell lines were significantly reduced (Fig. 1E). To further confirm the downregulation of FTO mRNA levels, we used immunohistochemistry assay to identify FTO protein expression in 68 PCa samples (Table 1), of which 24 have matching tumor tissues and corresponding paracancerous tissues. Consistently, based on the results of IHC, the FTO protein level in PCa tissues was significantly downregulated compared with adjacent tissues (Fig. 1F, G). Furthermore, the disease-free survival probability and progress-free survival were also analyzed by the UCSC Xena tool, which showed that lower FTO expression levels were correlated with a worse survival probability in PCa patients (*P* < 0.01) (Fig. 1H, I). In general, these data indicate that FTO expression is significantly lowered in PCa cells and tissues.

**Table 1:** Correlation between FTO expression and clinicopathological characteristics in prostate cancer.

Prostate Adenocarcinoma		*N*	FTO-High	FTO-Low	Chi squire	*p*
			17.65%(12)	82.35%(56)		
Age	> 65	56	10	46	0.0096	0.9218
	< =65	12	2	10		
Clinical stage	3–4	62	12	50	1.410	0.2350
	1–2	6	0	6		
Primary tumor	T3–T4	62	12	50	1.410	0.2350
	T1–T2	6	0	6		
Gleason score	< =6	5	2	3	1.855	0.1731
	> 6	63	10	53		

The *χ*^2^ test was used to test the association between categorical variables.

### FTO overexpression inhibits PCa proliferation and migration in vitro

**Fig. 2:**
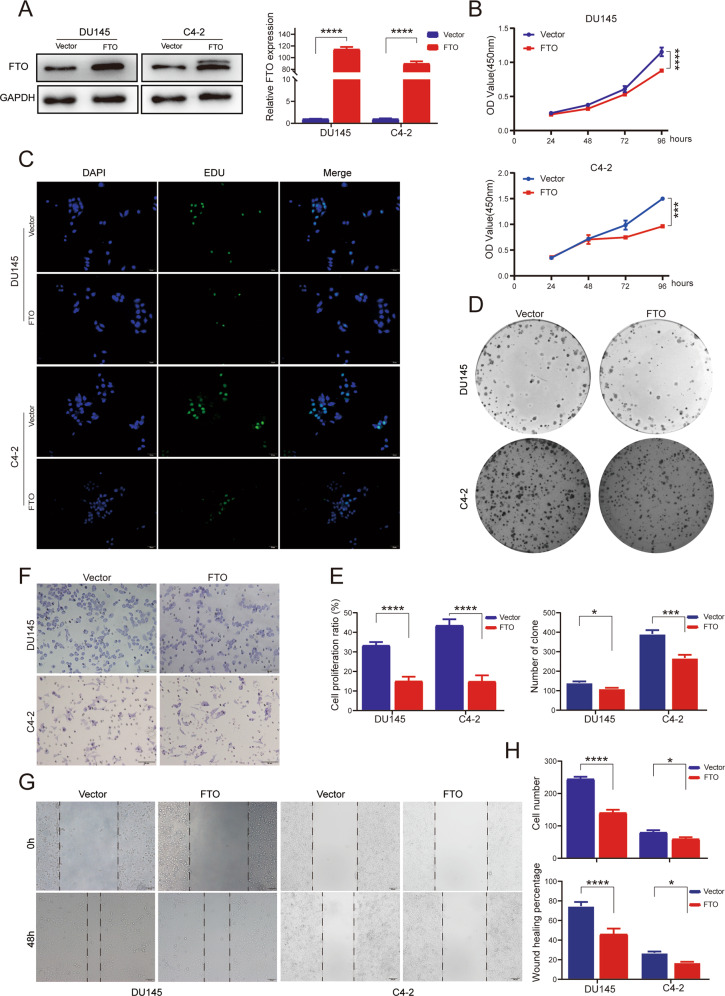
Overexpression of FTO inhibits the progression of PCa in vitro. **A** Western blot and RT-qPCR showed the overexpression efficiency of FTO plasmid and control vector in DU145 and C4-2 cell lines. GAPDH was the internal reference. **B** CCK-8 proliferation assay, **C** EdU assay, and (**D**) Colony-formation assay showed that up-regulating FTO inhibits PCa cells proliferation. **F** Transwell assay and (**G**) Wound healing assay suggested that overexpression of FTO significantly inhibits cells migration in DU145 and C4-2 cells. **E**, **H** Graphical illustration of statistical results of transfection of FTO plasmid or control Vector on cell proliferation and migration. *P < 0.05; ***P < 0.001; ****P < 0.0001; *t*-test. Error bars, mean ± SD (*n* = 3).

To investigate the potential characteristic of FTO in PCa, we conducted cell proliferation and migration associated assays. We constructed an FTO plasmid and vector control to transfect PCa cells. Compared with the control vector, FTO mRNA and protein expression levels in DU145 and C4-2 cells transient transfected with FTO-carrying plasmid increased (Fig. 2A). CCK8 assay showed that FTO overexpression significantly inhibited the cell growth of DU145 and C4-2 cells. We further verified the inhibitory effect of FTO overexpression on cell proliferation through EdU assay, and the overexpression of FTO dramatically lowered the colony formation rate in the colony formation test (Fig. 2B–E). Similarly, Transwell and Wound-healing assays showed that the overexpression of FTO significantly limits the ability of cell migration (Fig. 2F–H). Overall, according to these results, FTO performs a critical role in suppressing the proliferation and migration of PCa cells.

### Knockdown of FTO significantly promotes PCa progression in vitro

**Fig. 3:**
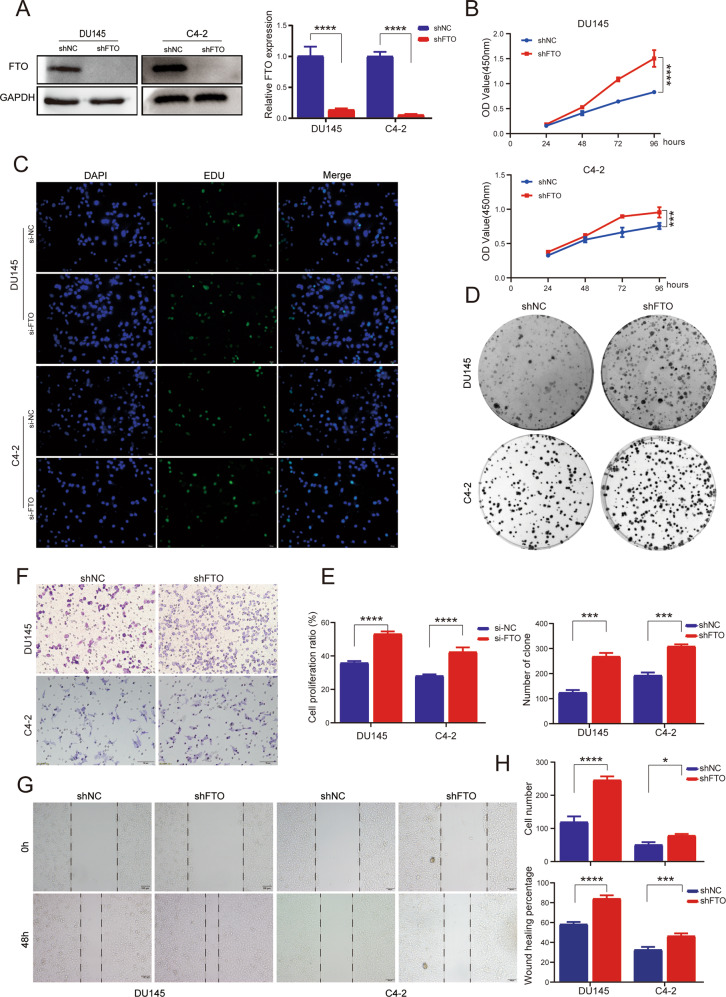
Knockdown of FTO promotes the PCa progression in vitro. **A** The knockdown efficiency of FTO with lentivirus constructs in DU145 and C4-2 cell lines was confirmed by western blot and RT-qPCR. GAPDH was the internal reference. **B** CCK-8 proliferation assay, **C** EdU assay. **D** Colony-formation assay were applied to detect the proliferation ability of PCa cells with FTO knockdown. **F** Transwell assay and (**G**) Wound healing assay showed that FTO knockdown markedly promotes cell migration in DU145 and C4-2 cells. **E**, **H** Graphical illustration of statistical results of transfection of FTO-shRNA or si-FTO on cell proliferation and migration. *P < 0.05; ***P < 0.001; ****P < 0.0001; *t*-test. Error bars, mean ± SD (*n* = 3).

To find out whether silencing FTO is essential for the growth of PCa cell, we stably knocked down FTO in DU145 and C4-2 cells by infecting specific shRNA lentiviruses. Western blotting and RT-qPCR assays were applied to confirm the effect of FTO-knockdown model (Fig. 3A). CCK8 and EdU assay proved that cell proliferation was remarkably increased after knocking down FTO. Moreover, the colony formation rate was faster than the control group (Fig. 3B–E). Furthermore, the cell migration capability was tested by Transwell and scratch healing experiments. Compared with control cells, downregulation of FTO increased the numbers of migrated cells and wound healing rate (Fig. 3F–H). We also analyzed the cell cycle and apoptosis of DU145 after silencing and overexpression of FTO by flow cytometry, and found that FTO did not affect the cell cycle and apoptosis of PCa (Supplementary Fig. 1). Overall, these results indicated that FTO may act as a tumor suppressor gene in PCa.

### Knockdown of FTO mediates CLIC4 mRNA degradation in an m6A-dependent way

**Fig. 4:**
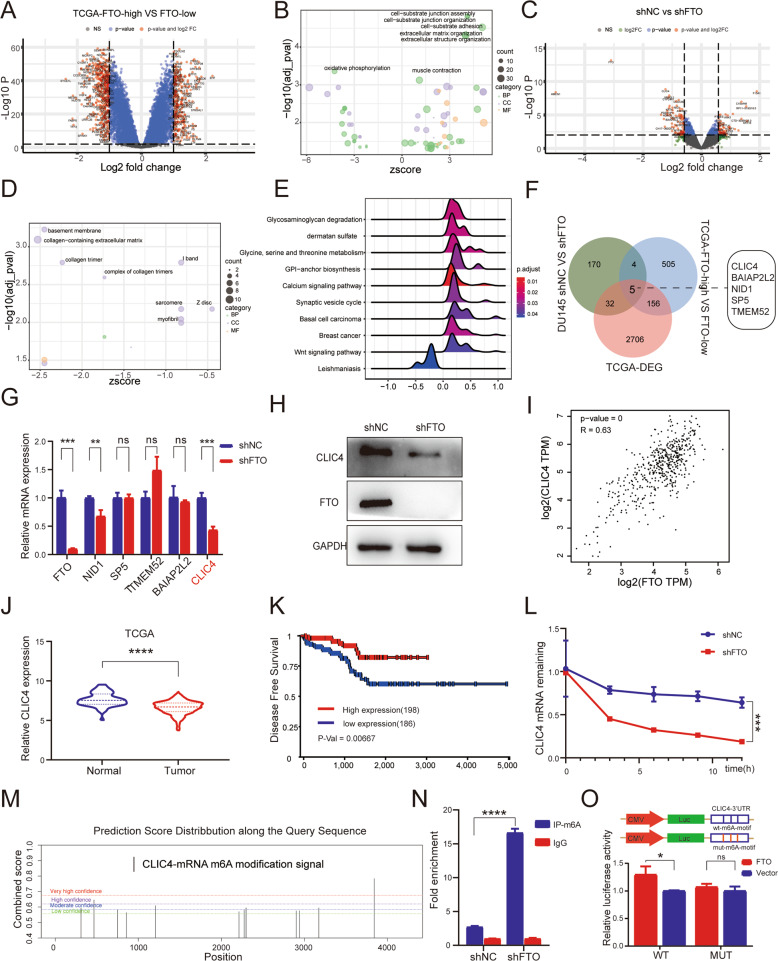
Knockdown of FTO promotes PCa progression by downregulated CLIC4. **A** Volcano plot of differentially expressed genes obtained from FTO high- and low-expression tumors. Significantly changed genes were plotted in red points. n.s Non-significant. **B** GO terms of significantly expressed genes with Bubble diagram. **C** Volcano plot of mRNA expression changes between shNC and shFTO group in DU145. **D** Bubble plot shows the GO terms of different genes between FTO knockdown and Control group. **E** The ridge plot shows the top 10 enrichment pathways of the differentially expressed genes in shNC and shFTO PCa cells by GSEA. **F** Venn diagrams for common genes from mRNA-seq, TCGA-DEG, and TCGA-FTO-high VS low genes. **G** CLIC4, BAIAP2L2, NID1, SP5, TMEM52, and FTO mRNA expression of DU145 shNC and shFTO cells were detected by qRT-PCR. **H** The effect of FTO knockdown on CLIC4 expression of DU145 cells was detected by western blot. **I** The scatter diagram was plotted to show the correlation between FTO and CLIC4 in TCGA PCa. **J** Violin plot shows the expression of CLIC4(log2(TPM + 1) in TCGA-PRAD. **K**, **L** Kaplan-Meier survival curve shows the disease-free survival time for different CLIC4 expression subtypes in PCa. **L** RT-qPCR showed the effect of FTO knockdown on the stability of CLIC4 mRNA in DU145. **M** The potential m6A sites of CLIC4 were predicted by SRAMP (Color lines of green, blue, purple, and red respectively represent low, moderate, high, and very high confidence). **N** MeRIP-qPCR was accepted to detect the m6A level alterations of CLIC4 after knocking down FTO in DU145. **O** Relative luciferase activities of pmirGLO-CLIC4-3′UTR with either wild type or mutant (A to C mutation) m6A sites after cotransfection with FTO plasmid or empty vector into HEK293T cell. *P < 0.05; ***P < 0.001; ****P < 0.0001; *t*-test. Error bars, mean ± SD (*n* = 3).

To clarify the possible molecular mechanism of FTO in the progress of PCa, the limma package was used to analyze the differentially expressed genes in the FTO-high group and the FTO-low group, and a total of 670 genes had significant changes. Gene Ontology (GO) evaluation confirmed that these genes were mainly concentrated at the cell-substrate junction assembly, cell-substrate junction organization, cell-substrate adhesion, extracellular matrix organization, and oxidative phosphorylation (Fig. 4A, B). All of these GO terms were critical to tumor metastasis and proliferation. Furthermore, to find out the potential direct target genes of FTO, we sequenced the RNA of DU145 cells after knocking down FTO. We obtained 211 genes that remarkably changed, and the GO terms were mainly involed in basement membrane, collagen-containing extracellular matrix, and collagen trimer (Fig. 4C, D), which were participated in cell migration. Besides, the top ten KEGG pathways enriched by GSEA indicated that FTO silencing was associated with PCa progression (Fig. 4E). Then we selected the common genes in TCGA-DEG, FTO-high vs FTO-low, and shFTO vs shNC (Fig. 4F). Among these five genes, CLIC4 altered most significantly in FTO-depleted cells (Fig. 4G). Western Blot also suggested that CLIC4 was downregulated after knocking down FTO (Fig. 4H). In addition, Pearson correlation analysis showed that CLIC4 expression is positively correlated with FTO in TCGA PCa dataset (Fig. 4I). Moreover, CLIC4 was downregulated in TCGA PCa dataset, which indicated a poor disease-free survival (Fig. 4J, K). What’s more, it has been reported that CLIC4 overexpression can inhibit the epithelial-mesenchymal transition in tumor cells [17]. According to this results, we speculated that CLIC4 might be a potential target of FTO.

To further investigate the probable mechanism underlying CLIC4 mRNA downregulation after FTO knockdown, we first compared the stability of CLIC4 mRNA in FTO stable knockdown cells and controls with Actinomycin D. Compared with the negative control, the mRNA decay rate of CLIC4 was significantly faster after FTO silencing (Fig. 4L). Since m6A is one of the most widespread mRNA modifications that can regulate the stability of RNA [5], and FTO is a major demethylation regulator of m6A, we studied the relationship between m6A modification and CLIC4 mRNA stability. First of all, we used the online website SRAMP to predict the potential m6A site of CLIC4, and we found several potential m6A modification sites on CLIC4 mRNA (Fig. 4M). Subsequently, MeRIP-qPCR assay was used to confirm the m6A methylation of CLIC4 mRNA in DU145 cells, and the m6A modification of CLIC4 mRNA was increased after knocking down FTO (Fig. 4N). To further verify that CLIC4 mRNA is a direct target of FTO-dependent m6A demethylation, we constructed both wild type and mutant CLIC4 3′UTR luciferase reporter. In the mutant sequence of CLIC4 3′UTR, the adenosine bases in m6A motif RRACH were substituted by cytosine. As expected, the luciferase assay showed that luciferase activity of wild type CLIC4 3′UTR-fused reporter was significantly increased after FTO overexpression, while the mutations showed no difference (Fig. 4O). To summarize, these outcomes demonstrated that m6A modification may be the reason of FTO-mediated CLIC4 mRNA expression.

### Silencing FTO promotes PCa proliferation and metastasis in vivo

**Fig. 5:**
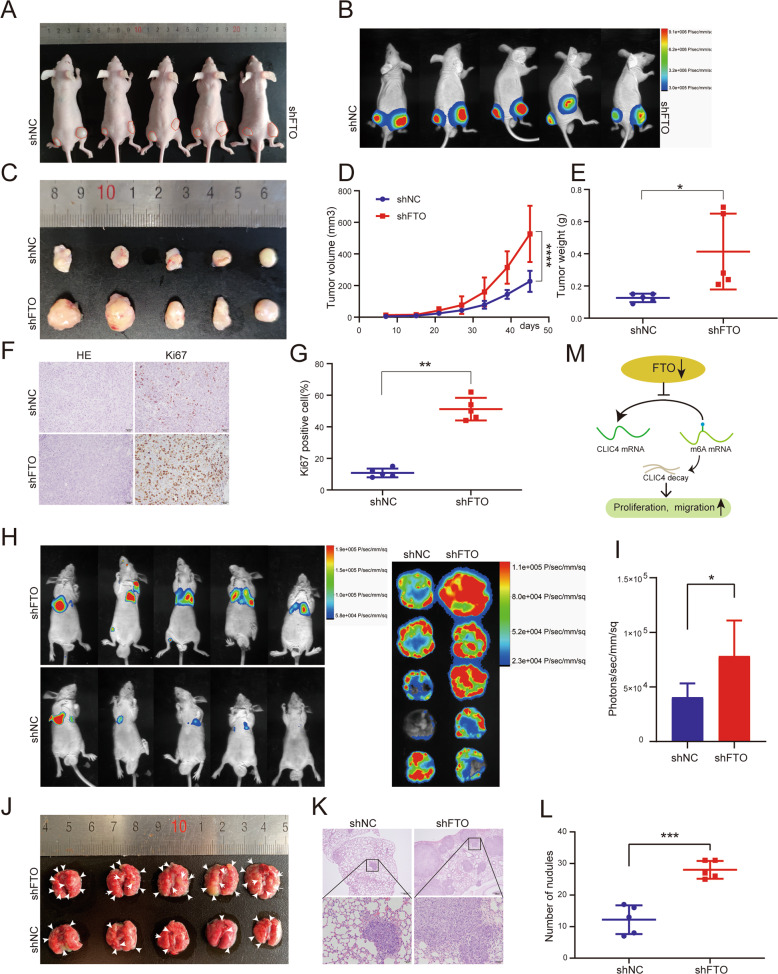
Knockdown of FTO promotes tumor growth and metastasis in vivo. **A**–**G** Subcutaneous tumor model. (**A**, **D**) The tumor growth curve of xenografts was plotted in the shNC and shFTO groups (*n* = 5). **B** The luciferase activities of subcutaneous tumor xenografts were measured with in vivo imaging system. **C** The BALB/c nude mice were sacrificed for the xenografts, and the size was measured by the beside ruler. **E** The anatomized subcutaneous tumor xenografts were weighed and analyzed with a student’s *t*-test between two groups. **F**, **G** Representative IHC staining micrographs of Ki-67 and HE staining in tumor xenografts were conducted. Scale bar = 50 μm. **H**, **L** Metastatic model (BALB/c nude mice). **H**, **I** The luciferase activities (radiance values) of lung metastasis were measured at 7 weeks by in vivo imaging system(*n* = 5). **J** Gross and microscopic illustrations of the lung to reconfirm the metastasis. **K** Representative H&E staining of metastatic organs, scale bar = 50 μm and 200 μm. **L** The statistic graphic shows the number of metastatic nodules in individuals. **M** Schematic diagram of the potential mechanism of FTO in PCa. Error bars, means ± SD; *P ≤ 0.05, ***P ≤ 0.001, ***P ≤ 0.0001.

To further validate the role of FTO in PCa, a subcutaneous implantation experiment was performed in male BALB/c nude mice to explore the effect of FTO-knockdown in PCa progress. FTO-knockdown DU145 cells were constructed by utilizing FTO shRNA with stably expressed luciferase, and subcutaneously injected into the mice (Fig. 5A, B). Results showed that the silencing of FTO effectively promoted the growth of prostate tumors, which was reflected in the significant increase in the volume and weight of tumor compared with the controls (Fig. 5C–E). The xenograft was then dissected and separated. H&E staining experiment was applied to show the histopathological characteristics of the tumor tissues. As seen by IHC staining, compared with the negative control, the expression of Ki-67 proliferation antigen in tumor tissues with FTO knockdown was significantly stronger (Fig. 5F, G).

We also constructed a tumor metastasis model by injecting FTO knockdown DU145 cells intravenously into mice to verify the effect of FTO on PCa metastasis. After 52 days, compared with the shNC group, the shFTO group observed an increase in lung metastasis sites, as reflected by the luciferase signal in the body. Then dissecting the lung organs and imaging them with in-vivo imaging system to further verify the metastasis (Fig. 5H, I). In addition, the lung metastasis was validated by histological examination (Fig. 5J–L). These experiments indicated that mice treated with shFTO cells generated more lung colonization than shNC cells. In summary, knockdown of FTO can dramatically promote tumor proliferation and metastasis in vivo.

## Discussion

Although it is reported that m6A modification is involved in different biological and pathological processes [18], the underlying mechanisms of m6A that participated in PCa progression remain largely unknown. Our results suggested an unusual tumor suppressor role in PCa, which is known as an oncogene in other types of tumors [19–21]. We demonstrated that FTO is downregulated in prostate adenocarcinoma, and the downregulation of FTO was negatively correlated with disease-free survival. Knocking down FTO markedly promoted the proliferation and migration of PCa cells in vitro. In the nude mice model, knockdown of FTO drastically motivated tumor proliferation and metastasis. In contrast, overexpression of FTO attenuated the malignant characteristics of PCa cells. Mechanistically, the reduction of FTO increases the m6A level of CLIC4 and then induces its mRNA attenuation, thereby promoting the progress of PCa. This evidence emphasized the valuable function of FTO in inhibiting the progression of PCa.

As a key mediator enzyme of m6A modification, FTO exerts a vital function in promoting tumorigenesis and anti-tumorigenesis in various cancers, depending on its context [13]. Overexpression of FTO was detected in AML, where it enhanced AML cell growth and transformation through regulating the m6A levels of ASB2 and RARA [22]. Recently, studies have showed that FTO can constrain ovarian cancer stem cells by promoting the decay of PDE1C and PDE4B mRNA [23]. In addition, (R)2-hydroxyglutarate (R-2HG), a cancer metabolite associated with mutant isocitrate dehydrogenases(IDH) in leukemias and gliomas, exerts anti-tumor effects by suppressing FTO and increasing total levels of m6A [24]. Interestingly, R-2HG was reported to be a tumor-promoted oncometabolite in PCa with IDH1 mutation. R-2HG can promote the m6A modification of TGFβ1 mRNA, further promote the production of circRNA-51217, and increase PCa cell invasion through the miRNA-646/TGFβ1 and p-Smad2/3 axis [25]. Whereas, the critical role of FTO in PCa has not yet been clarified. Consistent with this, our research showed that FTO depletion was associated with an increase in m6A levels, thereby increasing the tumorigenicity and metastasis of PCa, while FTO overexpression showed the opposite effect. These provided convincing evidence for FTO as a tumor suppressor in PCa.

Chloride intracellular channel 4(CLIC4) is mentioned to be a direct transcriptional target of p53 and c-Myc, which can inhibit cell proliferation through the TGFβ signaling pathway [26–28]. Besides, it serves as a tumor suppressor in gastric cancer and cutaneous squamous cell cancer, as manifested by knockdown CLIC4 facilitates tumor cells migration and invasion [17, 29]. Previous studies have shown a declined expression of CLIC4 in PCa tissues compared to paired normal tissue [30]. Combining with our bioinformatics analysis, we showed that CLIC4 was downregulated in the PCa tissues, and the low expression of CLIC4 is associated with a poor prognosis. Further experiments indicated that CLIC4 may be regulated by FTO via epigenetic modification. Although we have proved that FTO inhibits the progress of PCa, our understanding of the mechanism of FTO in PCa is still limited. For instance, we have not yet completely identified the specific m6A readers that degrade the mRNA of CLIC4. In addition, considering the fact that CLIC4 is a target of p53 and c-Myc [26], more evidences are needed to confirm the relationship between these molecules and FTO. In general, we will continue to explore the underlying mechanism of FTO in the future.

To summarize, our results indicate that FTO is downregulated and is closely related to the poor prognosis of PCa. Functionally, FTO inhibits PCa cells proliferation and metastasis both in vitro and in vivo. The depletion of FTO increases the m6A levels of CLIC4, leading to the attenuation of its mRNA (Fig. 5M). Therefore, our findings provide evidence for FTO as a prognosis biomarker and therapeutic target for PCa.

## Materials and methods

### PCa samples and clinical information

PCa samples, formalin-fixed paraffin-embedded, were collected from Zhujiang Hospital from 2015 to 2018. The detail data of the patients were listed in Supplementary Table 1. The patient had signed an informed consent to participate in the study following the ethical protocols of the Ethics Committee of Zhujiang Hospital, Southern Medical University.

### Cell culture and cell lines

Human PCa cell lines and one immortalized prostatic epithelial cell line, including PC-3, C4-2, DU145 and RWPE-1, purchased from Stem Cell Bank, Chinese Academy of Sciences. All cell lines were identified by STR profiling. These cancer cells were cultured in RPMI 1640 medium with 10% fetal bovine serum (South America origin, IC-1905, USA), and RWPE-1 was cultured in Keratinocyte Serum-Free Medium (KSFM) (Gibco, No. 10744-019) with 5 ng/mL EGF (epidermal growth factor) (Gibco, No. 10450-013) in a humidified environment containing 5% CO_2_ at 37 °C.

### Reagents and transfection

FTO plasmid vector was assembled by inserting the full-length cDNA of FTO into vector CV702(pCMV-MCS-3FLAG-SV40-Puromycin), further validated by sequencing, the empty and lentivirus vector were purchased from GeneChem (Shanghai, China). siRNA (small interfering RNA) of FTO and negative control (NC) were purchased from RiboBio (Guangzhou, China). Transfection reagent Lipo3000 was purchased from Invitrogen (L3000015) for transfection of the plasmids and siRNA on the basis of the manufacturer’s suggestion. Cell lines that stable knockdown of FTO were established with the lentivirus vector based on the manufacturer’s instructions. And the cells transfected with lentivirus were selected in puromycin (2 μg/ml) for one week. Sequence for short hairpin RNA (shRNA) and siRNA for FTO were 5′- GACCUUCCUCAAGCUCAAUGA -3′.

### RNA isolation and quantitative RT-PCR (RT-qPCR)

TRIzol (Invitrogen, USA) and Advantage RT-for-PCR kit (Takara, Japan) were used to extract total RNA and perform reverse transcription according to the manufacturer’s requirement. TB Green PCR Master Mix (Takara) and Applied Bio-systems 7500 Fast Real-Time RCR System (United States) were adopted for qPCR assay. Three independent assays were performed to collect data, and GAPDH was regarded as internal control. Primers sequences of this study were shown in Supplementary File 1.

### Western blot assay

RIPA lysis buffer (#KGP250, KeyGEN, China) was used to extract protein. And 25 µg protein was separated by SDS-PAGE gel (10%, Bio-Rad) and transferred to a Polyvinylidene fluoride (PVDF) membrane (Millipore Germany). Then blocked the membrane with 5% fat-free milk for an hour and incubated overnight at 4 °C with these primary antibodies: anti-FTO (#14386 S, CST), anti-GAPDH (#5174, CST), anti-CLIC4 (#A7088, ABclonal). Next, incubate the membrane with the secondary antibody (anti-rabbit/mouse IgG-HRP (#7074 S/#7076 S, CST) in blocking buffer for 1 h, shaking gently at room temperature. Subsequently, incubate the membrane with ECL substrate and visualize with TANON system (Shanghai, China). Image J 1.46(National Institutes of Health) was supported to detect the intensity of protein bands. Raw data were shown in Supplementary File 2.

### Cell viability

Cell viability was quantified with Cell Counting kit-8 (CK-04, Dojindo) following the Producer’s instructions. Briefly, incubated PCa cells in 96-well plates (2000 cells/ well). Next, added 10% CCK-8 to each well, and incubated for 2 h in a 37 °C incubator. Finally, microplate reader (EXL800, BioTek Instruments) was applied to detect the optical density (OD) at 450 nm.

### Colony formation

DU145 and C4-2 were incubated into a six-well plate (500 cells/well), and incubated for an appropriate time to form colonies. Subsequently, the colonies were fixed in 4% paraformaldehyde and then dyed with Giemsa (#DM0007, Leagene). Three independent replicates were performed in each experiment.

### EdU incorporation assay

PCa cells proliferation activity was analyzed with Cell-Light EdU Apollo488 In Vitro Kit (#C10310-3, Ribobio) following the manufacturer’s statement. Then, the cells were pictured with a microscope (Olympus, Japan). Finally, the ratio of EdU stained PCa cells (green fluorescence) to Hoechst labeled cells (blue fluorescence) in each well was measured.

### Transwell migration assays

5 × 10 ^ 4 cells, resuspended in 300 μl FBS-free medium, were inoculated into the upper chamber of the insert (8 mm pores; Corning, United States), 500 μl complete medium containing 10% FBS was put into the lower chamber. The cells were fixed with 4% paraformaldehyde for 20 min after an appropriate incubation period, and then stained with Giemsa. Finally, the randomly selected fields were snapped with an inverted microscope (Olympus IX71) at 200 × magnification. The number of cells in each image were calculated and each test was repeat three times.

### Scratch healing assay

PCa cells were cultured in 6-well plates. After growing to 90%, a linear wound was made with a 10 μl pipette tip and cleaned the cell debris with PBS. Next, culturing the cells in serum-free medium. At the designated time point, the wound was monitored and photographed with an inverted microscope with a magnification of 100 times. The scratch-healing areas were measured by Image J.

### Cell cycle analysis

FTO stable knockdown cells DU145 and controls were seeded in 6-well plates (2 × 10^5/well). After 48 h, cells were harvested and the Cell Cycle Detection Kit (#KGA512, Keygen) was used to detect the cell cycle. In short, cell pellet was washed twice with cold PBS, and fixed with cold 70% ethanol at 4 °C overnight. Then wash twice with cold PBS buffer, and incubated with 100 µl RNase A at 37 °C. Following, stained with 400 µl PI at 4 °C for 30 min and analyzed with a FACS Calibur flow cytometer.

### Cell apoptosis assay

The apoptosis cells were detected by using Cell Cycle and Apoptosis Analysis Kit (#C1052, Beyotime). Briefly, 2 × 10^5 cells DU145 were cultured in 6-well plate. After 48 h of FTO plasmid transfection, the culture medium and cells were collected. Centrifuge around 1000 g for 3 min, and wash twice with cold PBS buffer. Subsequently, resuspend the cell pellet in 100 μl Annexin binding buffer, add 5 μl PI (propidium iodide) and 5 μl Annexin V-FITC, and incubate in a dark room at room temperature for 15 min. Finally, 400 µl binding buffer was added and tested by FACS Calibur flow cytometer within an hour.

### RNA-seq

RNA sequencing experiment and data analysis were conducted by Seqhealth Technology Co., LTD (Wuhan, China). In short, total RNAs were obtained from cells using Trizol. According to the producer’s instructions, use KCTM Stranded mRNA Library Prep Kit for Illumina to prepare a stranded RNA sequencing library. Finally, Novaseq 6000 sequencer (Illumina) with PE150 model was applied to sequence. And edgeR package was used to identify the differentially expressed genes. *P*-value < 0.05 and absolute fold-change > 1.5 were considered statistical significance.

### Bioinformatics analysis

Publicly available PCa tissues data was downloaded from The Cancer Genome Atlas (TCGA). FTO expression based on Gleason score was analyzed by the UALCAN website. The differentially expressed genes between PCa and normal samples (TCGA-DEG) were calculated with edgeR package on R platform (R 4.0.2). We split the tumor cases into two groups based on FTO expression. The upper-25%(FTO-high, FTO positively correlated) had the highest FTO expression level, while the lower-25%(FTO-low, FTO negatively correlated) had the lowest FTO expression level. The limma package was used to compare differentially expressed genes, FDR < 0.01 and LogFC >1 or < -1 was the cutoff line. Gene Ontology and Gene-set enrichment analysis were carried out with the ClusterProfiler package [31]. The GSE6919 dataset was downloaded from the NCBI GEO and analyzed with the limma package. The potential m6A target site was predicted by the online tool SRAMP [32].

### Measurement of mRNA stability

FTO stable knockdown cell lines (shFTO) and controls (shNC) were treated with the transcription inhibitor actinomycin D (5 μg/ml) at indicated times before cell collection. Trizol was used to capture total RNA and RT-qPCR to quantify the relative levels of target mRNAs, and 18 S was used as an internal control.

### Luciferase reporter assays

The DNA fragment of CLIC4 3′UTR containing m6A-spercific sites was inserted into pmirGLO reporter vector (GeneCopoeia, Guangzhou, China). The mutant CLIC4-3′UTR sequence was replaced the adenosine bases within the m6A motif sequences to cytosine, further verifying by DNA sequencing. The detailed CLIC4 3′UTR wild-type and mutant sequences were provided in Supplementary File 1. After cotransfecting HEK293T with 200 ng luciferase and FTO plasmids into 24-well plate for 48 h, the luciferase activities were measured with Luc-Pair™ Duo-Luciferase HS Assay Kit (#LF001, GeneCopoeia).

### Animal experiments

The 4-week-old male BALB/C nude mice were provided by the Southern Medical University Animal Center (Guangzhou, China) and raised with Specific Pathogen Free (SPF) conditions. Approximately 2 × 10^6 PCa cells (DU145 transfected with shFTO and shNC) were injected subcutaneously in mice. The tumor volume (V = (0.5*length*width^2)) was measured with Vernier caliper every week. Ten mice were randomly divided into two groups, 2 × 10^6 cells transfected with shFTO and shNC were resuspended with 100 μl PBS and injected into the mouse tail vein to create a metastatic model. After 7 weeks, the mice were anesthetized, and D-luciferin (#D-Luciferin, Apexbio) was injected intraperitoneally, then used the IVIS imaging system (Caliper Life Sciences) to visualize the luciferase signal. IHC and hematoxylin-eosin (H&E) staining were used to further detect the characteristics of xenograft tumors and lung metastasis. The animal researches were conducted in accordance with institutional guidelines approved by the Zhujiang Hospital, Southern Medical University.

### Immunohistochemical analysis (IHC)

IHC was performed using PV-6000-6.0 kit, purchased from Zhong Shan Golden Bridge Biotechnology (Beijing, China), primary antibodies Ki-67 (Abcam, #16667) and FTO (Abcam, #ab92821) according to the manufacturer’s suggestion. The result of IHC was analyzed by three independent pathologists. The scores of positive cells are as follows: 0 (staining range, <5%), 1 (5–25%), 2 (26–50%), 3 (51–75%), or 4 (>75%). Intensity scores were designated as 0 (no staining), 1 (weakly, light yellow), 2 (moderately, tan), and 3 (strongly, brown). The product of the two scores was used as the final score. A score of >6 was considered high expression, and a score < =6 was considered low expression.

### Methylated RNA Immunoprecipitation and RT-qPCR

All specific operations were conducted in accordance with the instruction of the BersinBioTM Methylated RNA Immunoprecipitation (MeRIP) Kit (BersinBio, #Bes5203). Briefly, total RNAs were extracted from PCa cells (approximately 2 × 10^7) with trizol, and resuspended with 850 μL IP buffer. Except for 50 μL of the total RNA as input, the rest of RNA was applied to immunoprecipitation with IgG and m6A antibody at 4 °C overnight, respectively. The prepared Magnetic Beads of Protein A/G were incubated with IgG and m6A-specific antibody in IP buffer at 4 °C for an hour, then digested the beads with Proteinase K at 55 °C for 45 min. After that, the RNA in the supernatant was extracted with Phenol-Chloroform-Isoamylol. Finally, the RNA was quantified by Nanodrop 2000 (Thermo Scientific), and further performed qRT-PCR. The primers were listed in Supplementary File 1.

### Statistical analysis

Statistical analyses of this study were performed using GraphPad Prism8.03 (GraphPad Software, La Jolla, USA). The two-tailed Student’s t-test was recommended for the data analysis between two groups whose data follow a normal distribution. For data that did not conform to the normal distribution, the Mann–Whitney test was accepted. The log-rank test was used to calculate Kaplan–Meier survival curve. One-way analysis of variance (ANOVA) was utilized to analyze continuous variable groups. Statistical significance was defined as a P value less than 0.05.

## Supplementary information

Supplementary File 2

Supplementary Figure 1

Supplementary File 1

Supplementary Table 1

## Data Availability

The raw data supporting the results of this study will be made available by the authors, without undue reservation.
