# Supplementary figures and images for: N6-methyladenosine demethylase FTO suppressed prostate cancer progression by maintaining CLIC4 mRNA stability
Source: Cell Death Discov. 2022 Apr 9;8:184. doi: 10.1038/s41420-022-01003-7 (PMC8994758; doi:10.1038/s41420-022-01003-7)

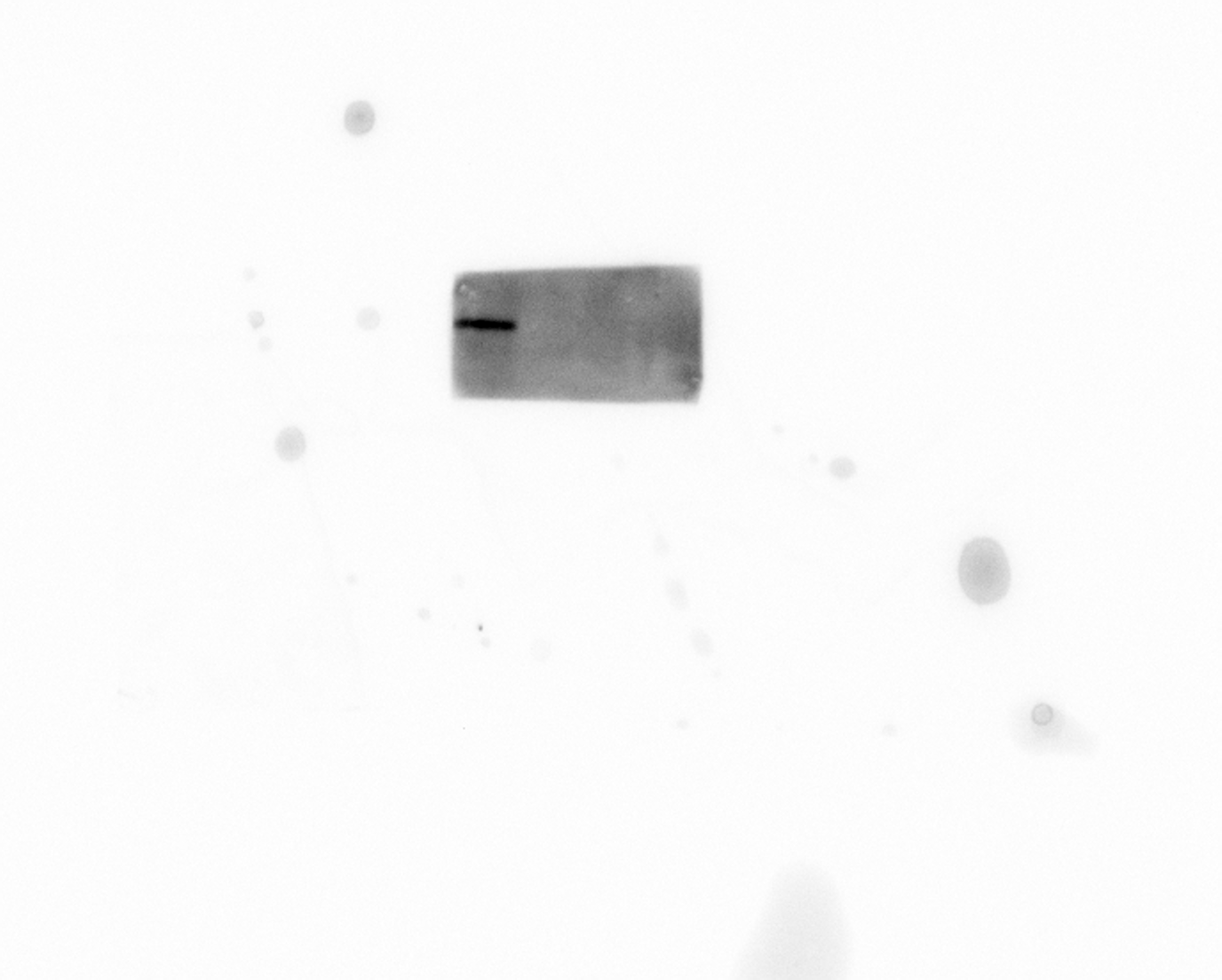

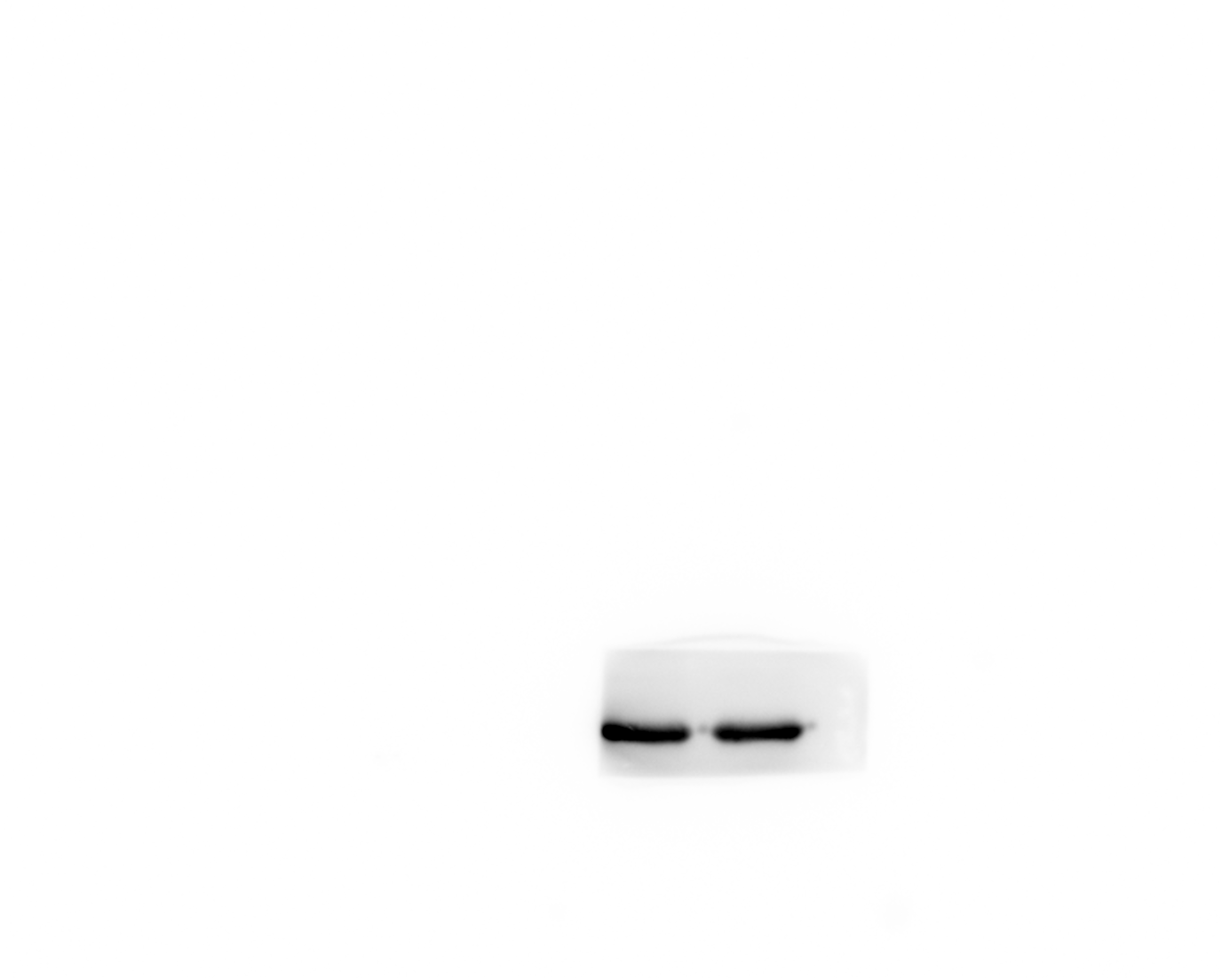

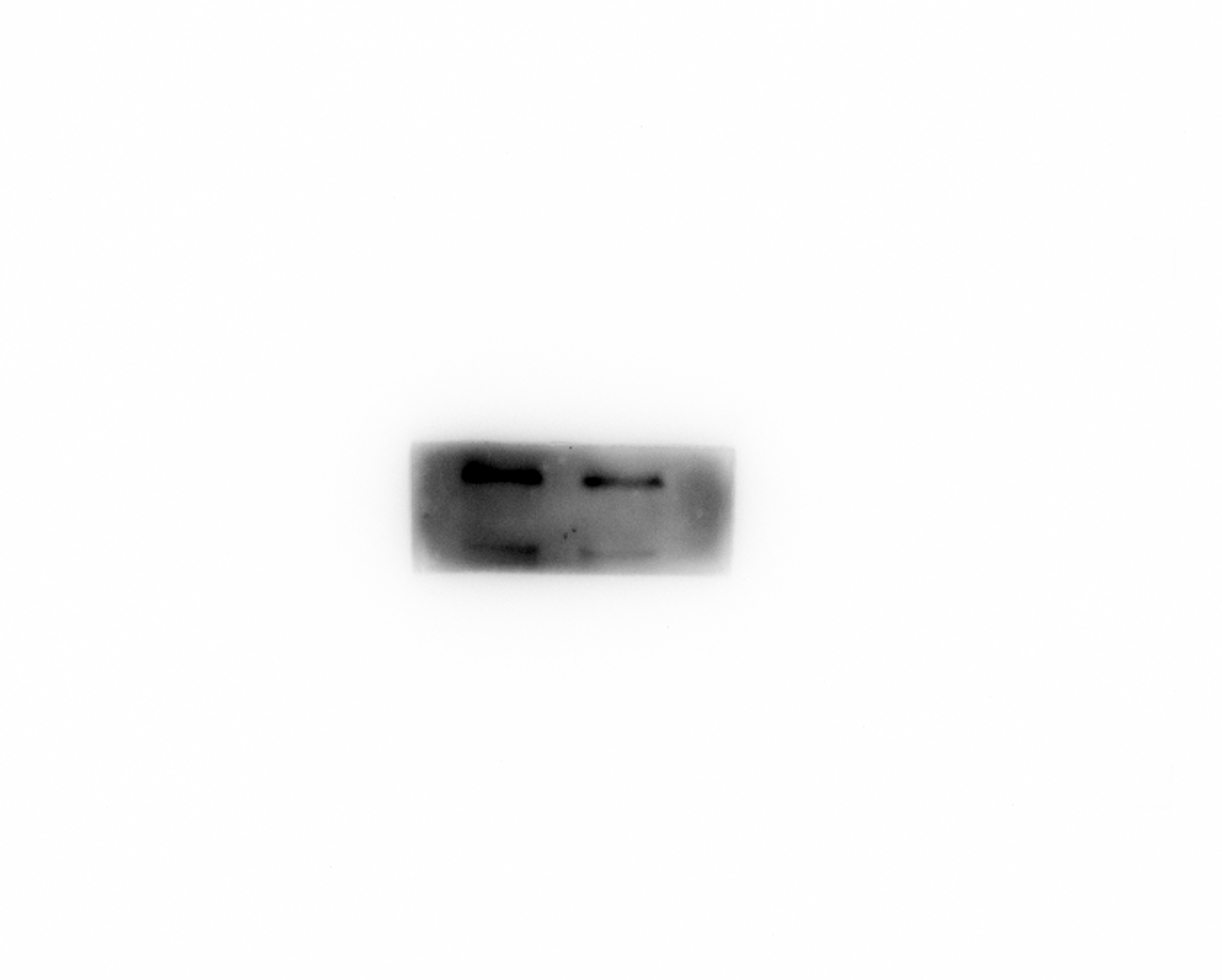

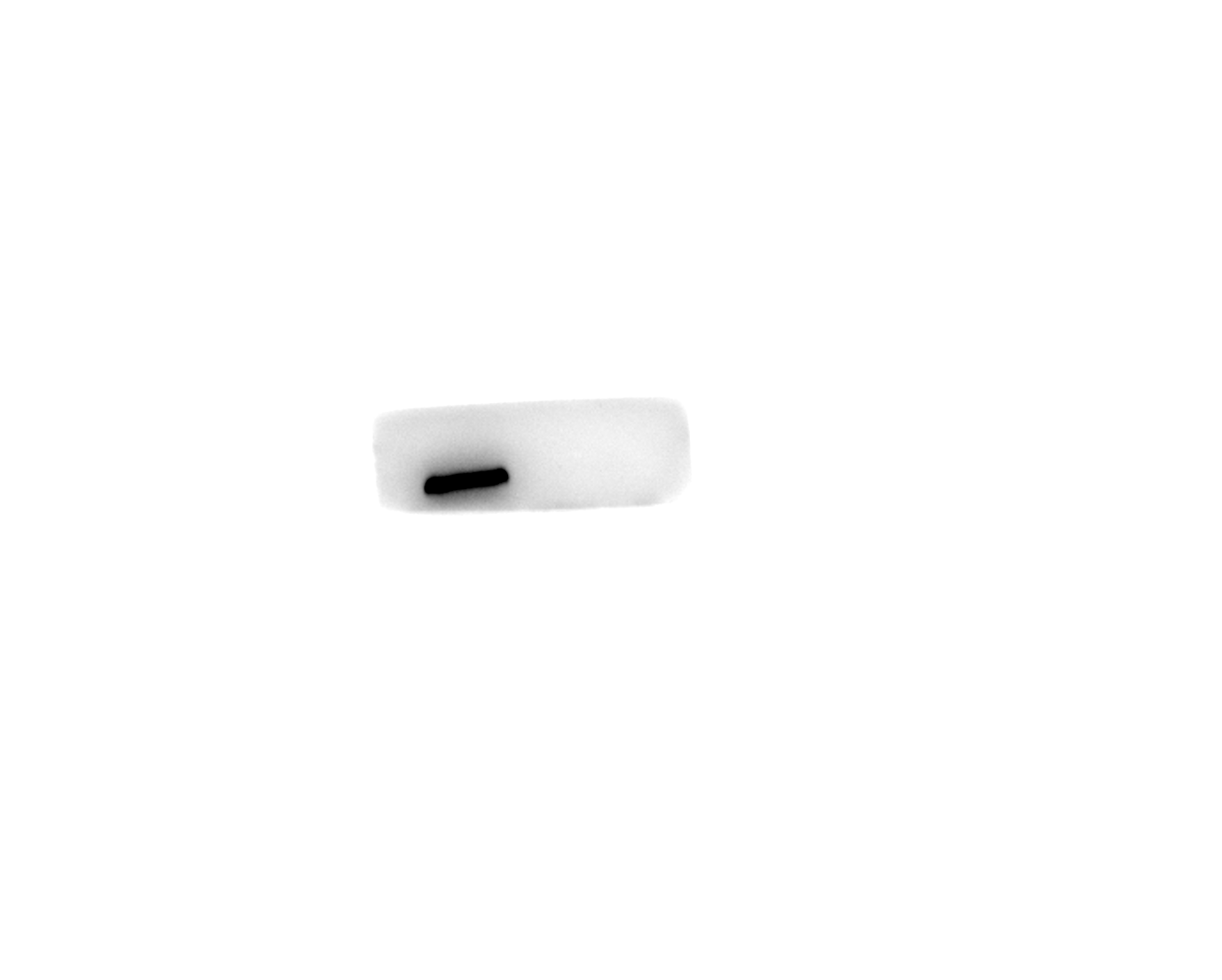

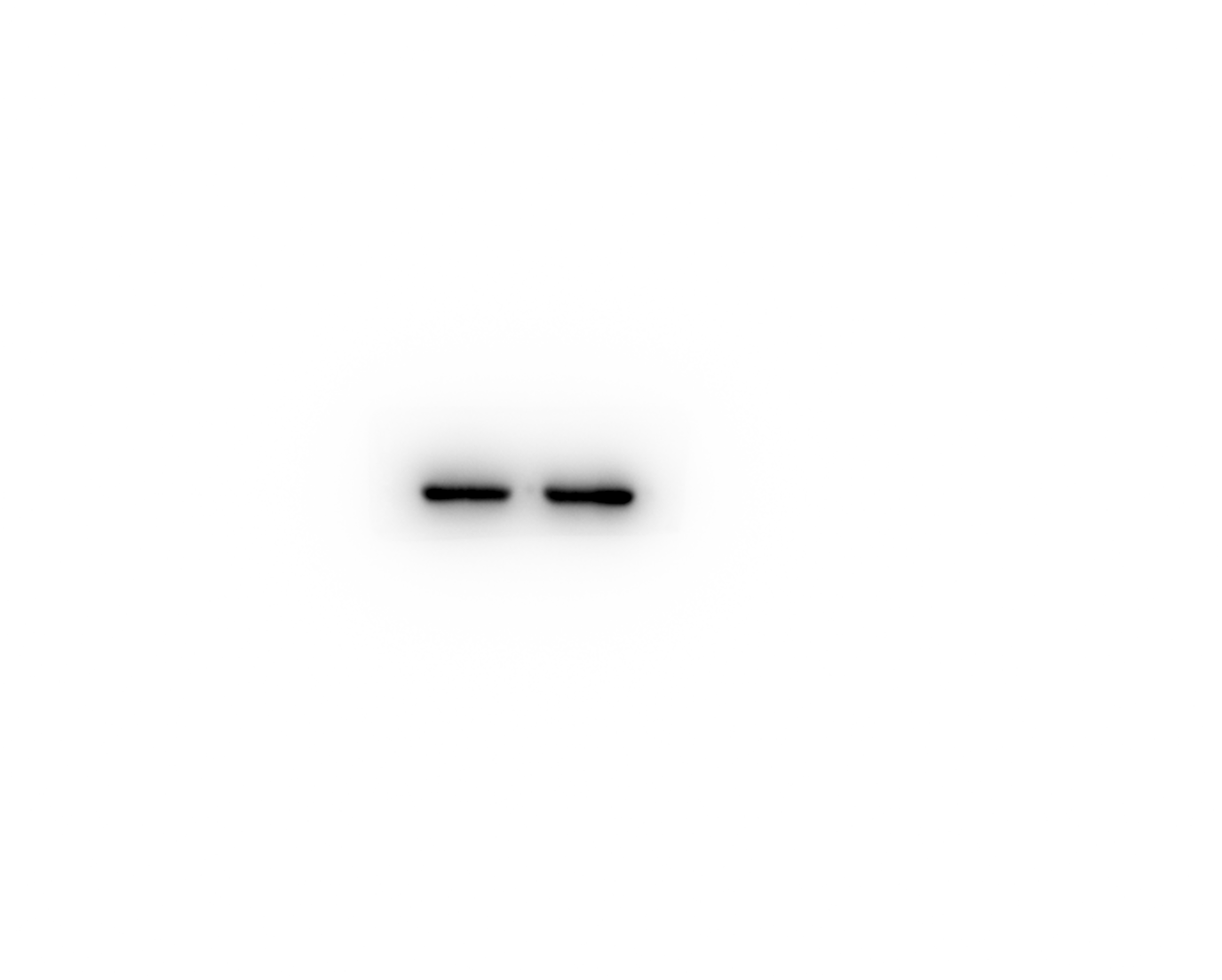

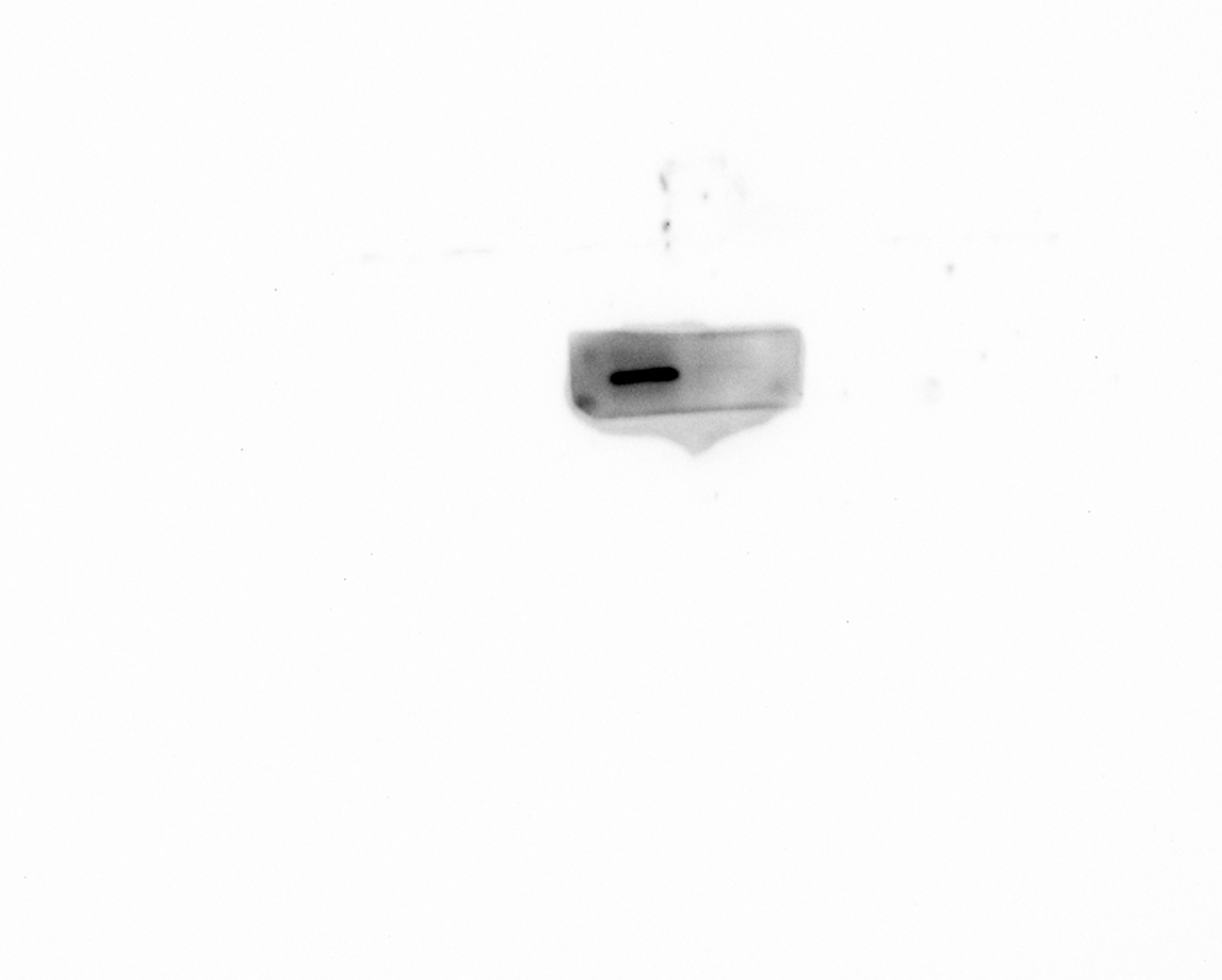

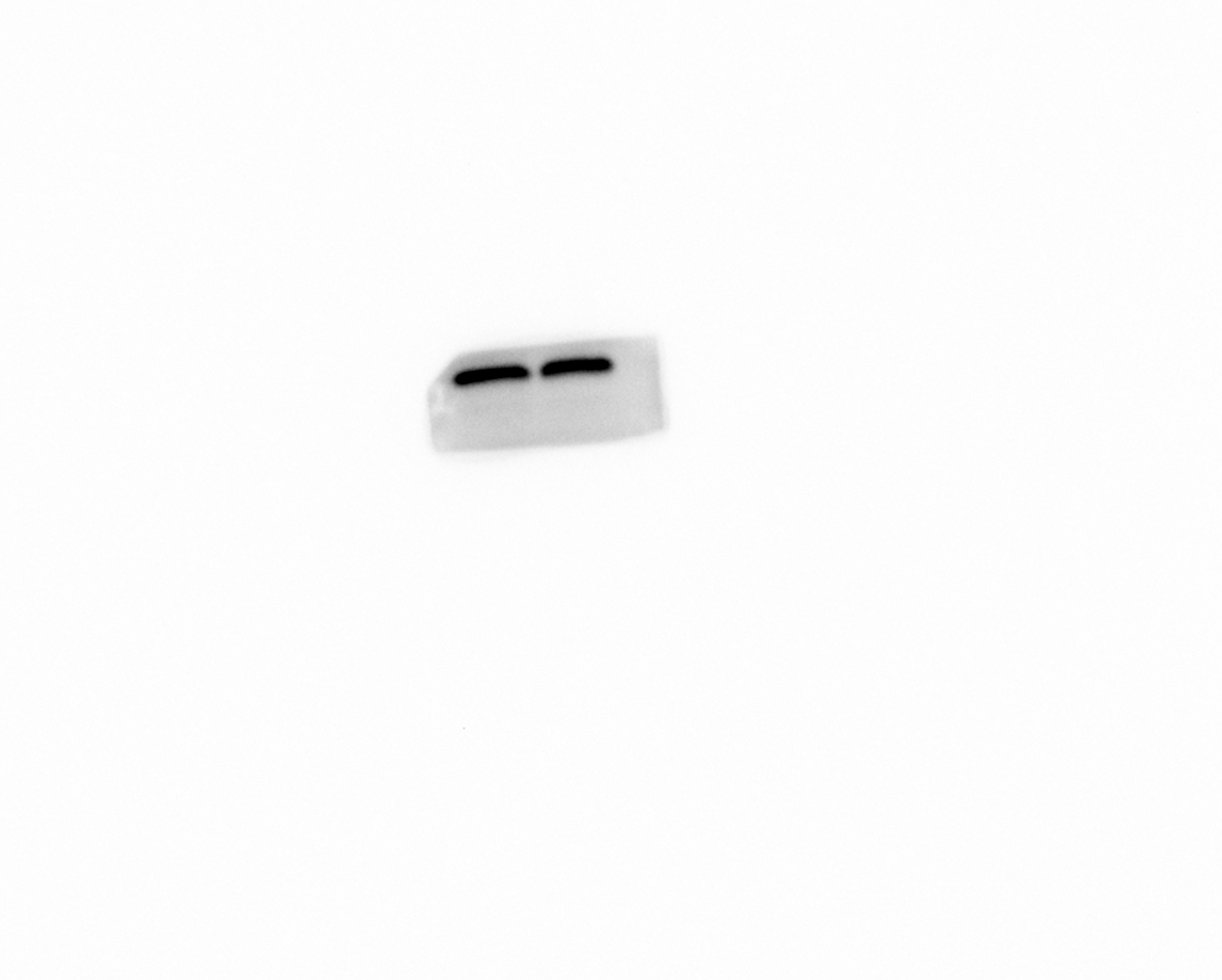

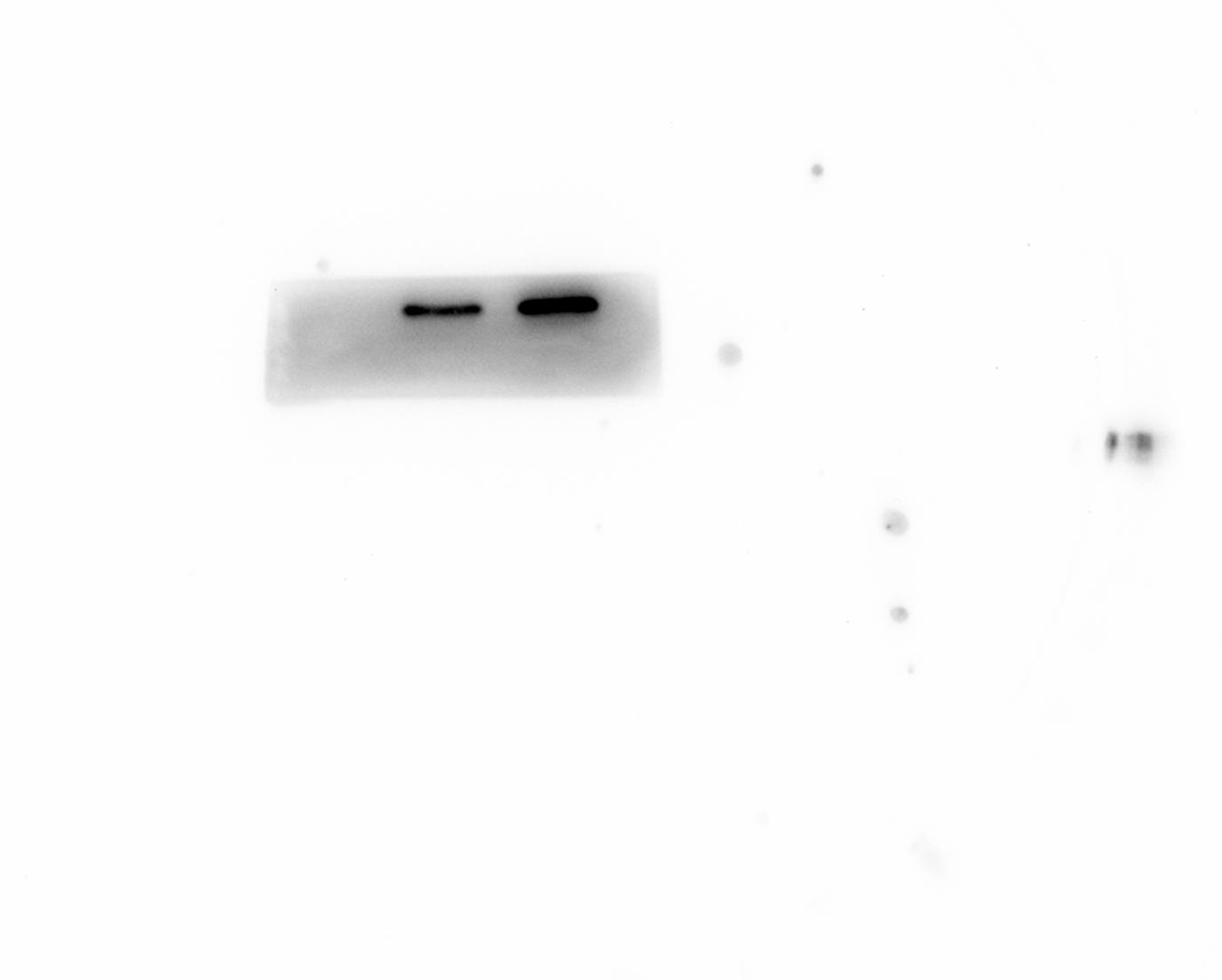

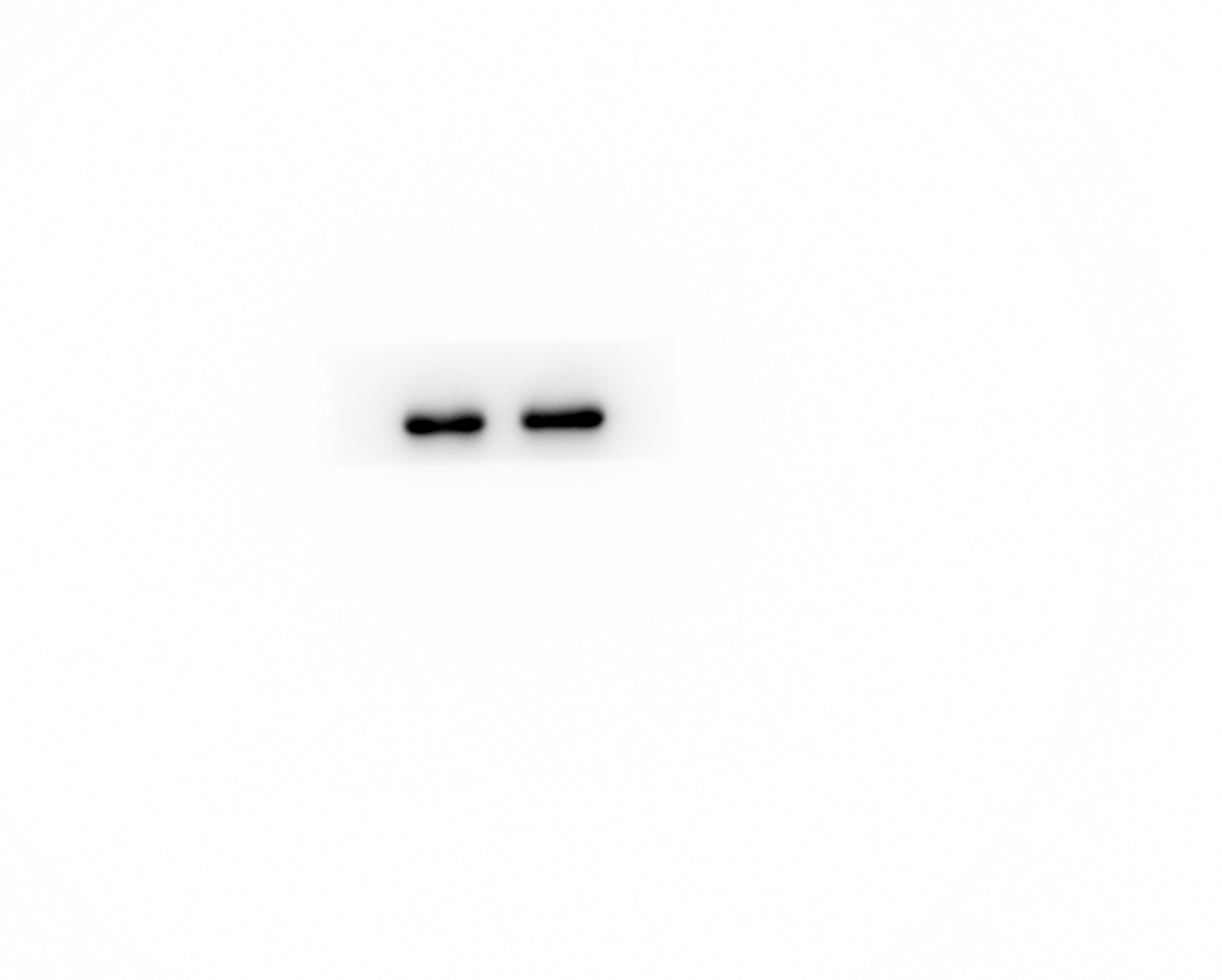

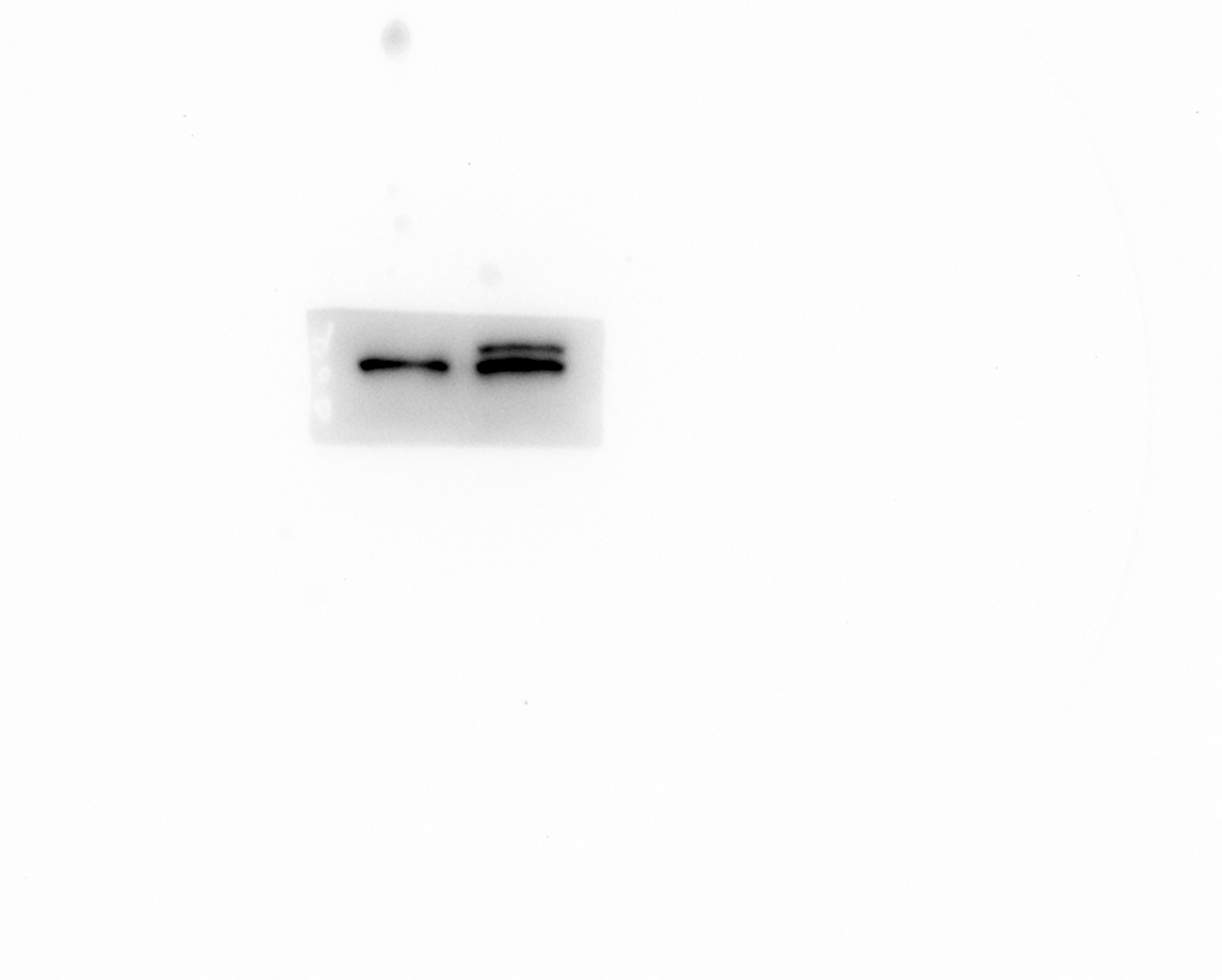

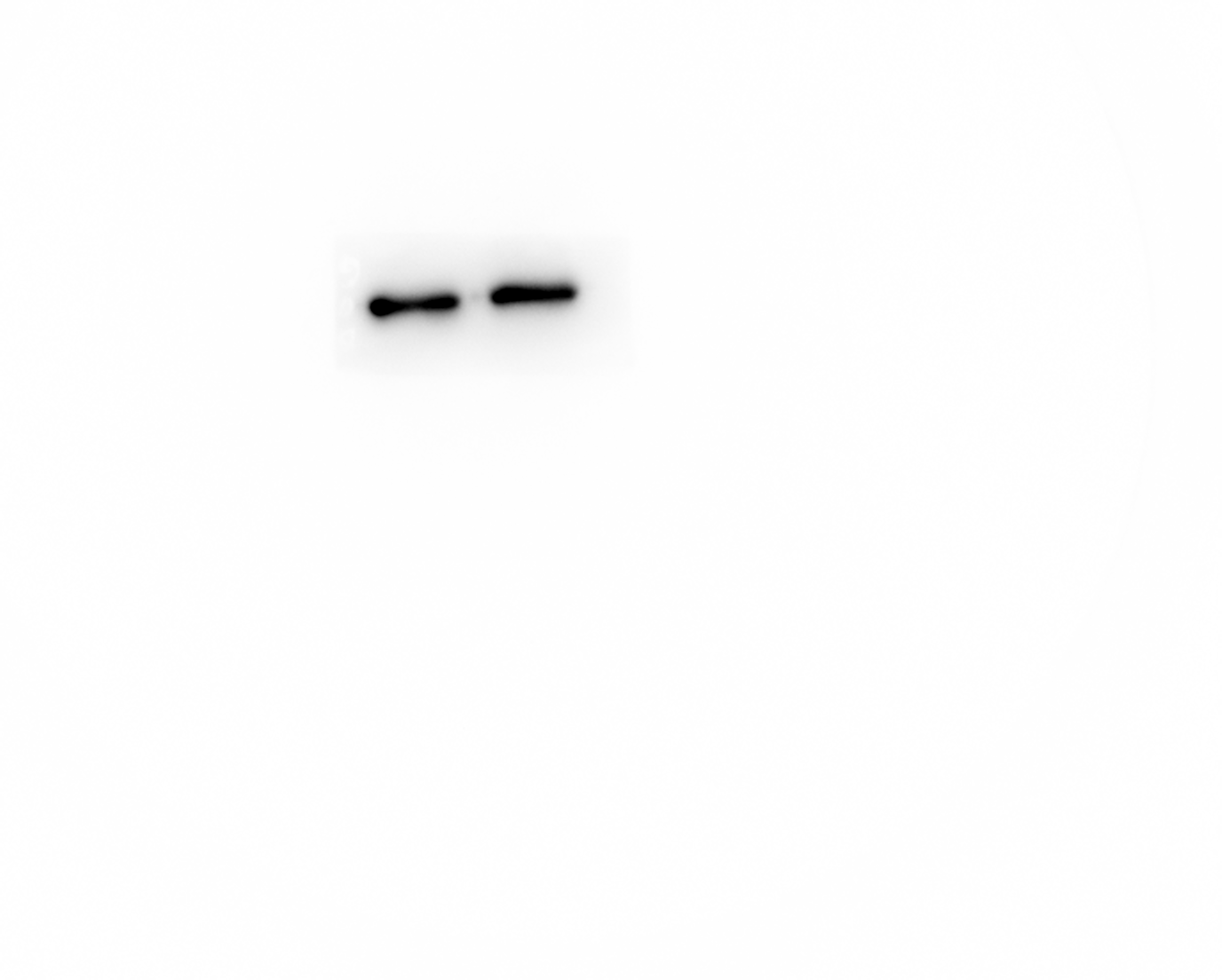

Supplement: Supplementary file 1 — Supplementary File 2 [file 41420_2022_1003_MOESM1_ESM.docx]

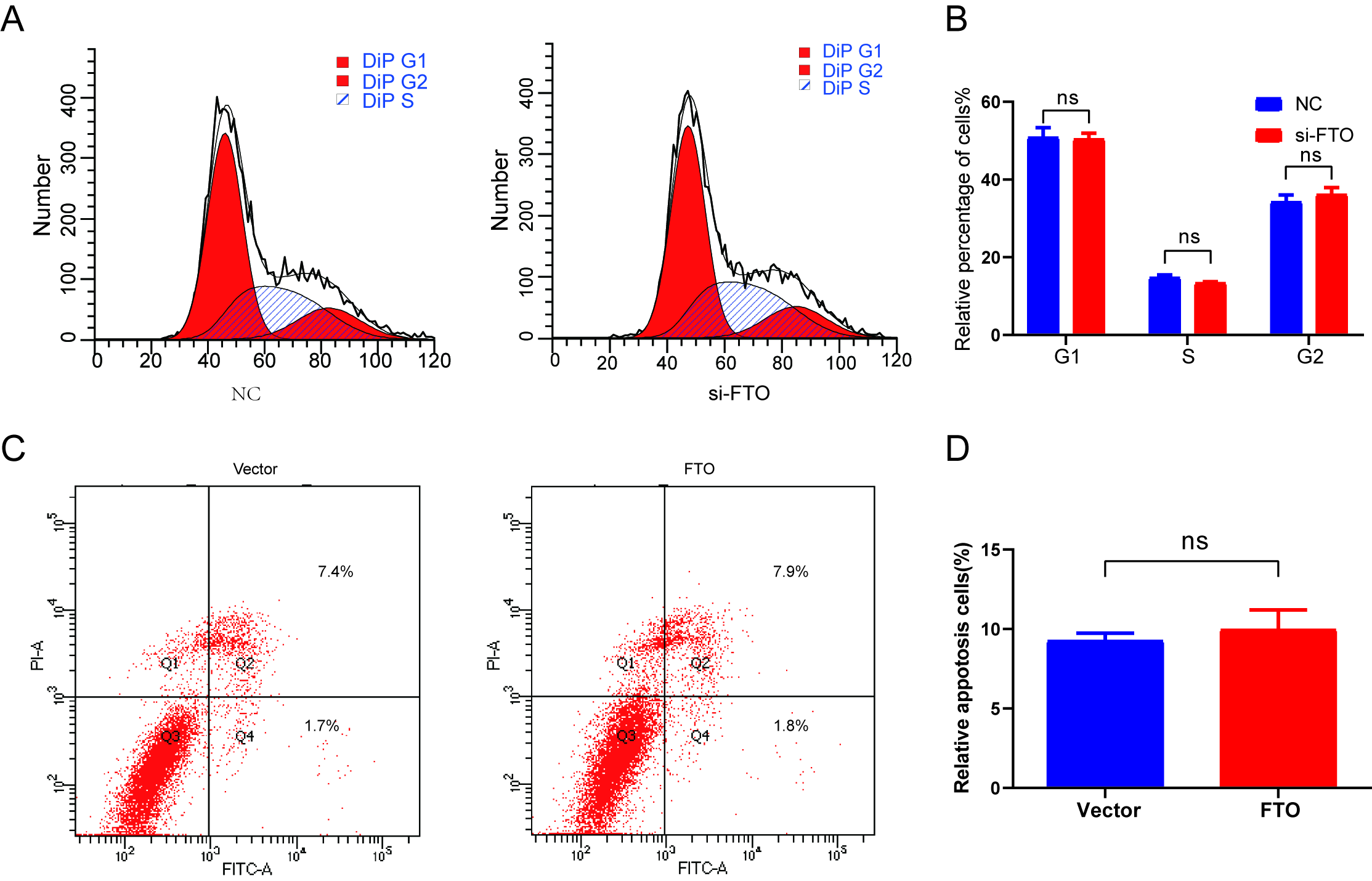

Supplement: Supplementary file 2 — Supplementary Figure 1 [file 41420_2022_1003_MOESM2_ESM.tif]
