# Supplementary material for: N6-methyladenosine demethylase FTO suppressed prostate cancer progression by maintaining CLIC4 mRNA stability
Source: Cell Death Discov. 2022 Apr 9;8:184. doi: 10.1038/s41420-022-01003-7 (PMC8994758; doi:10.1038/s41420-022-01003-7)
Supplement: Supplementary file 3 — Supplementary File 1 [file 41420_2022_1003_MOESM3_ESM.docx]

primers

FTO Forward primer ACTTGGCTCCCTTATCTGACC

FTO Reverse primer TGTGCAGTGTGAGAAAGGCTT

GAPDH Forward primer CCAGGTGGTCTCCTCTGACTTC

GAPDH Reverse primer GTGGTCGTTGAGGGCAATG

CLIC4 Forward primer AAGAAGTCTTATGCCCTCCCA

CLIC4 Reverse primer TCTCCAGTGCTTCATTAGCCT

NID1 Forward primer GACACAGTTCACCTGCGAGT

NID1 Reverse primer GAAAACTGGTAGCCCTCCACA

SP5 Forward primer ACTCAGGGGTCACTACAGGA

SP5 Reverse primer AAACCCGACCGCTTAGTACA

TMEM52 Forward primer TCACAGCTGGTTGTGTCCG

TMEM52 Reverse primer CCATAGGGATGACTGCCACG

BAIAP2L2 Forward primer GCCGAGGTCTACTTCAGTGC

BAIAP2L2 Reverse primer GCTGGGTGTCAGACATCTGC

YTHDF2 Forward primer CTCTTGGAGCAGTACAAAATGGA

YTHDF2 Reverse primer GACCAAGCAGCTTCACCCAA

18SrRNA Forward primer ACACGGACAGGATTGACAGA

18SrRNA Reverse primer GGACATCTAAGGGCATCACA

***CLIC4*-3'UTR with wild-type m6A sites:**

GTAGTTTTGCCTGATCTCACTCATTGCACTTCCTGGAGTTAAATTTTCCAACAGCCATGTTGAGGAATAGCACTCTGCATGTTTTTGTTTTGTTTTTCGGGGTTTTTTTTAATTGAAGCCCTAAACCAGGAATTATTTGTGTTCTAACAGGAGGATGAACTTGCTGAAAATAAAACTTTGCTATGTATTTACTCTTTTTTAAAAGACAAAAGCAAAACCAGACTTTCTACGTACTACTCCAAAGACTGTGATTGTGACTATAATACATTTTTGGTAATTTTTTTATACCTAATTTGTATAGGAAGTGCTATTTCTCATAGGCTGTTTCTTGAAATTTTAAGTTTATTGCTTTAAAATGGCAGTGTTTCTCCCACTTTGATATGCTAACATTTA

***CLIC4*-3'UTR with mutant m6A sites:**

GTAGTTTTGCCTGATCTCACTCATTGCACTTCCTGGAGTTAAATTTTCCAACAGCCATGTTGAGGAATAGCACTCTGCATGTTTTTGTTTTGTTTTTCGGGGTTTTTTTTAATTGAAGCCCTAAACCAGGAATTATTTGTGTTCTAACAGGAGGATGA**C**CTTGCTGAAAATAAAACTTTGCTATGTATTTACTCTTTTTTAAAAGACAAAAGCAAAACCAG**C**CTTTCTACGTACTACTCCAAAG**C**CTGTGATTGTGACTATAATACATTTTTGGTAATTTTTTTATACCTAATTTGTATAGGAAGTGCTATTTCTCATAGGCTGTTTCTTGAAATTTTAAGTTTATTGCTTTAAAATGGCAGTGTTTCTCCCACTTTGATATGCTAACATTTA

**Supplementary Figure 1**. FTO does not affect the cell cycle and apoptosis of PCa. (A) Representative FACS readout of cell cycle after knocking down FTO in DU145. (B) Quantification of PI staining depicted in Fig.S1 A (n = 3). (C&D) Cell apoptosis was analysis by Flow cytometry after FTO was overexpressed in DU145 (n=3). Error bars, means ± SD.
